# Cetacean Poxvirus in Two Striped Dolphins (*Stenella coeruleoalba*) Stranded on the Tyrrhenian Coast of Italy: Histopathological, Ultrastructural, Biomolecular, and Ecotoxicological Findings

**DOI:** 10.3389/fvets.2018.00219

**Published:** 2018-09-11

**Authors:** Cristiano Cocumelli, Gianluca Fichi, Letizia Marsili, Matteo Senese, Giusy Cardeti, Antonella Cersini, Enrica Ricci, Fulvio Garibaldi, Francesco Scholl, Giovanni Di Guardo, Giuliana Terracciano

**Affiliations:** ^1^Istituto Zooprofilattico Sperimentale Delle Regioni Lazio e Toscana, Rome, Italy; ^2^Veterinary Practitioner, Pisa, Italy; ^3^Department of Physical Sciences, Earth and Environment, University of Siena, Siena, Italy; ^4^Department of Earth, Environment and Life Sciences, Università di Genova, Genoa, Italy; ^5^Facoltà di Medicina Veterinaria, Università di Teramo, Teramo, Italy

**Keywords:** poxvirus, environmental pollutants, immunosuppression, *Stenella coeruleoalba*, Mediterranean Sea, Italy, Striped dolphin

## Abstract

Tattoo skin disease (TSD) is a poxviral disease typical of cetaceans. Two juvenile and well-preserved male striped dolphins (*Stenella coeruleoalba*), found stranded along the Tuscany and Latium coasts of Italy in 2015 and 2016, respectively, showed typical skin lesions ascribable to TSD. Histological, ultrastructural and biomolecular investigations confirmed a poxviral aetiology for the aforementioned skin lesions. To our knowledge, this should be the first report of TSD in cetaceans stranded along the Italian coastline. As organochlorines like PCBs and DDTs are known to be highly immunotoxic, the tissue loads of these contaminants were evaluated, in order to increase our knowledge on their potential role as well as on the relationships between the level of exposure to these pollutants and poxviral infection's occurrence.

Tattoo skin disease (TSD) is a poxviral cetacean disease. Poxviruses are large DNA viruses infecting humans and several domestic and wild animal species (1, 2). Limited available sequencing of Cetacean Poxvirus (CePV) isolates places them in an unclassified genus within the *Chordopoxvirinae* subfamily, which includes at least two groups: *Cetacean Poxvirus type 1* (CePV-1) in odontocetes and CePV-2 in mysticetes (3). Six distinct CePV-1 clusters infecting different families of odontocetes have been hitherto identified, with some Authors suggesting they could belong to six CePV species (CePV-1 to 6) (4).

In several cetacean species, TSD has been extensively reported, being observed in free-ranging odontocetes from the North Atlantic, the Eastern Pacific, and the Mediterranean Sea (5). Notwithstanding the above, there are very few TSD descriptions in cetaceans from the Mediterranean area, primarily based on photo-identification data (2, 6, 7), with no reports in striped dolphins and other cetaceans of different ages and sex which were necropsied during an unusual mortality event in 2013 along the Tyrrhenian coast of Italy (8). Juveniles are reported to have a higher prevalence of TSD lesions than adults, although in free-ranging cetaceans with poor health conditions adults tend to show an increased TSD prevalence (5).

It has been additionally suggested that anthropogenic factors, such as prolonged exposure to polychlorinated biphenyls (PCBs), may play a major role in the emergence of skin diseases in harbor porpoises, presumably because of the immunosuppressive effects of these compounds (5, 7). Odontocetes, which are top predators, are among the animal species most exposed to the toxic effects of PCBs. Marine mammals have been also found to have an extremely low capacity to metabolize organochlorine (OC) compounds compared to birds and terrestrial mammals (9, 10), making them particularly vulnerable (11).

The present work reports the first identification of TSD in two striped dolphins (*Stenella coeruleoalba*) stranded along the Italian coastline, along with the biomolecular characterization of the CePV strains detected and the results of ecotoxicological analyses on the two TSD-affected dolphins under study.

On March 12, 2015, a juvenile male striped dolphin, 170 cm in length and 45 Kg in weight (case 1), was found stranded on the Tyrrhenian coastline (Western Mediterranean Sea) of Tuscany, near Marina of Massa (municipality of Massa), Italy (44.007764, 10.097706). On January 20, 2016, another juvenile male striped dolphin, 150 cm in length and 35 Kg in weight (case 2), was found stranded on the Tyrrhenian coast of Latium, in Anzio (municipality of Rome) (41.452888, 12.618507). The body condition score showed average to poor values for both animals, with a blubber thickness at dorsal fin level of 20 mm in case 1 and 9 mm in case 2. At *post mortem* examination, both dolphins appeared to be in a good preservation *status* (code 2/5), according to Geraci and Loundsbury (12).

Upon external examination, the two animals showed peculiar skin alterations. In case 1, these were characterized by multiple, 5–20 cm-wide, coalescing lesions, with stippled lightly gray foci surrounded by dark edges and affecting the animal's head, behind the blowhole, around the left eye (Figure 1) and on the lateral aspect of the mandible; in case 2, a single, 20 mm-wide, round, yellowish lesion with a slightly dark edge was observed on the skin of the mandibular region.

**Figure 1:**
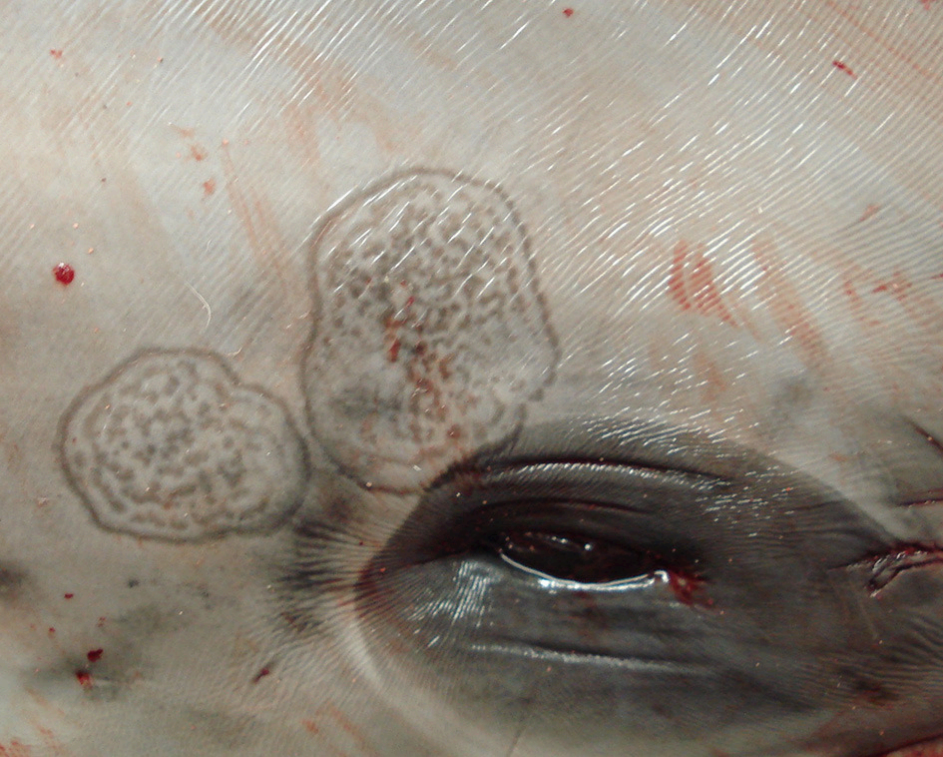
Skin lesions. *Stenella coeruleoalba*. (case 1). Wide, multifocal to coalescing, lightly gray areas with dark edges and stippled in the center, affecting the head.

Samples of the skin lesions from both animals, along with tissue samples from the major organs, were fixed in 10% neutral buffered formalin for histopathology and frozen for both electron microscopy and biomolecular investigations, as reported elsewhere (4). After paraffin embedding, skin tissue blocks were cut into 4 μm-thick section, which were routinely stained with haematoxylin-eosin (HE), to be finally observed under a light microscope. In order to evaluate viral morphology, the cutaneous lesions were processed for analysis via electron microscopy. Frozen portions of the skin lesion were homogenized by mortar with sterile sand and suspended 1:1 in bidistilled water. The sample was then negatively stained with sodic salt of phosphotungstic acid (NaPT) 2% and finally observed with a transmission electron microscope (TEM) (Philips, EM 208) (4).

Polymerase chain reaction (PCR) was additionally conducted on further frozen skin portions of the lesion, following the procedure described by Barnett et al. (4). More in detail, a 543 bp-long segment of the viral polymerase gene (DNApol gene) was sequenced and analyzed using BioEdit Sequence Alignment (13). Phylogenetic trees were generated with neighbor-joining method using SEAVIEW v4.2 (14) and the confidence levels were calculated using bootstrapping (2,000 replicates) as described by Barnett et al. (4). Furthermore, in order to deepen our knowledge on the putative role of persistent environmental pollutants in the onset as well as in the pathogenetic evolution of the infection, analyses for hexachlorobenzene (HCB), dichlorodiphenyltrichloroethanes (DDTs) and PCBs were duly performed following the method of the U.S. Environmental Protection Agency (EPA) 8081/8082, with laboratory modifications (15). The extracted organic material (EOM%) from freeze-dried blubber, muscle and liver, was calculated in all samples. Capillary gas-chromatography revealed op'- and pp'- isomers of DDT and its derivatives DDD and DDE, as well as about 30 PCB congeners. Total PCBs were quantified as the sum of all congeners. These congeners constituted 80% of the total peak area of PCBs in the biopsy. Total DDTs were calculated as the sum of *op'*DDT, *pp'*DDT, *op'*DDD, *pp'*DDD, *op'*DDE, and *pp'*DDE. The results were expressed in ng/g dry weight (d.w.). Canonical variables (CAN) of DDTs + PCBs were calculated as described by Marsili et al. (16). The central nervous system, the lymphoid tissues and the lungs of both animals were also examined by means of PCR for pathogens known to be related with immunosuppression (Dolphin Morbillivirus, DMV and Herpesvirus, HV) as well as for additional pathogens of major concern for cetaceans (Toxoplasma gondii and Brucella ceti). Adequate positive and negative controls were used in each run against the 4 aforementioned pathogens, with the former ones being represented by tissues from DMV-, HV-, Toxoplasma gondii-, and Brucella ceti-infected striped dolphins.

Microscopic observation of skins samples from both dolphins revealed a multifocal, severe, hydropic degeneration of *stratum spinosum* keratinocytes, with simultaneous occurrence of round, 5–20 μm in diameter, eosinophilic, glassy structures (intracytoplasmic eosinophilic inclusion bodies) compatible with B-type poxviral inclusions (Guarnieri bodies) (Supplementary Materials). The overlying *stratum corneum* was mildly hyperplastic (1.5 times normal) and in case 1 with heavily hyperpigmented keratinocytes, occasionally containing Guarnieri bodies (Figure 2). The spleen and the lymph nodes of both striped dolphins had a diffuse increase in their lymphoid follicle number (lymphoid hyperplasia), with a moderate to severe reduction in the number of cells in the germinal centers (lymphoid depletion) and deposition of small amounts of a hyaline material consistent with amyloid deposition; in case 2, a mild scattered apoptosis of single lymphocytes was also detectable. Numerous virus particles (~240 × 150 nm), similar in size and ovoid shape to parapoxviruses, but with a disordered surface structure typical of CePV, were observed by means of TEM in cutaneous lesion specimens from both dolphins.

**Figure 2:**
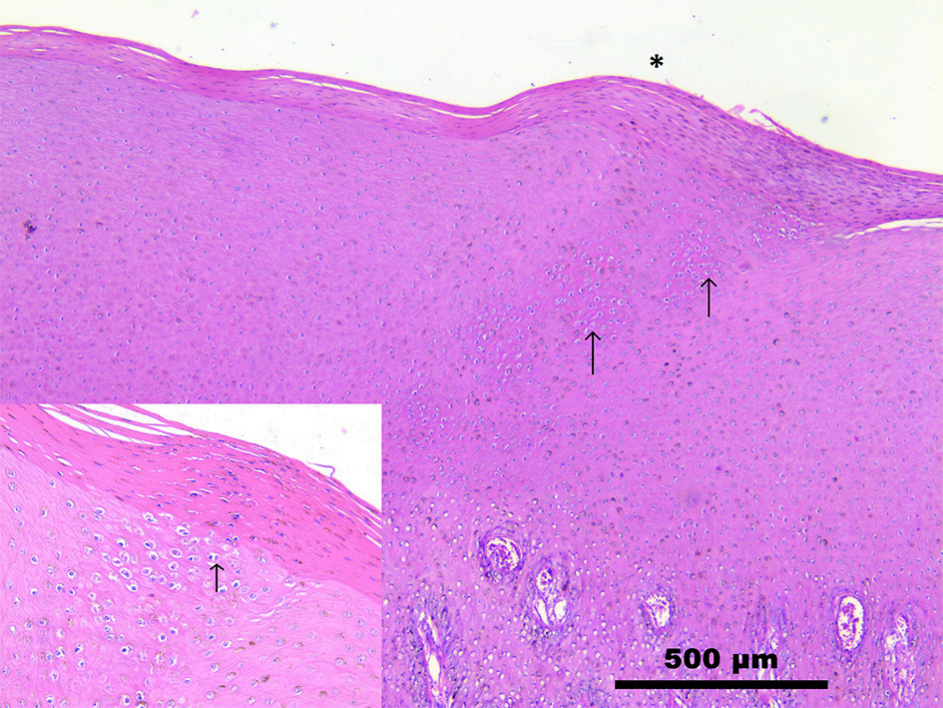
Skin. *Stenella coeruleoalba*. (case 1). Mild increase in the thickness of *stratum corneum* (*), with hyperpigmentation and severe hydropic degeneration of keratinocytes (inset). Numerous round, up to 25 μm, eosinophilic glassy structures (intracytoplasmic inclusion bodies) surrounded by a clear halo (Guarnieri bodies) are visible (arrows). See also Supplementary Materials. Scale bar = 500 μm.

The presence of Poxvirus-specific DNA was demonstrated both in case 1 (GenBank accession number KY652339) and in case 2 (GenBank accession number KY652340), with 100% and 99.63% homology, respectively, with a CePV-1 isolate obtained from a harbor porpoise (*Phocoena phocoena*) found stranded along the coast of the United Kingdom (GenBank accession number KC409040.1). The genomic identity value between the two CePV-1 strains recovered from the two herein investigated dolphins was 98.90%.

With respect to phylogenetic analyses, the two strains clustered in the same group identified as CePV-5 (Figure 3) by Barnett et al. (4). According to Barnett et al. (4) and based upon the currently available knowledge on CePV genus, the results herein reported do not allow us to identify the real number of CePV species and if there are either a species-specificity or any spatio-temporal relationships between the strains previously recognized as belonging to CePV-1 cluster and those characterized from the two herein investigated striped dolphins.

**Figure 3:**
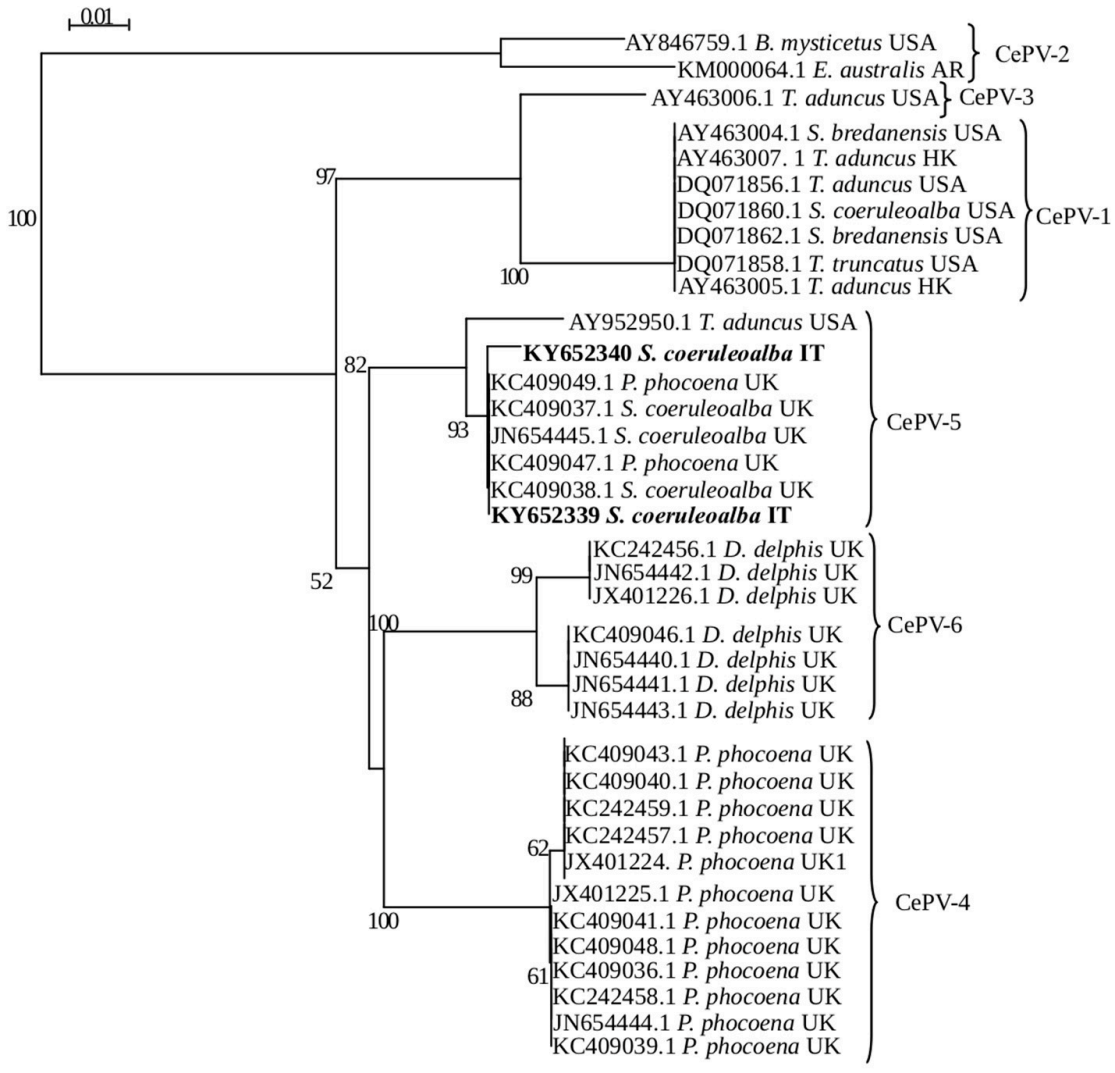
Phylogenetic analysis of CePV polymerase gene sequences available in GenBank. Each sequence name comprises the GenBank accession number, host species and geographic origin. The groups identified by Barnett et al. (4) are indicated by braces. Phylogenetic trees generated with neighbor-joining method using SEAVIEW v4.2 and the confidence levels were calculated using bootstrapping (2,000 replicates). Bootstrap values under 50% were omitted.

Furthermore, laboratory results testing for presence of DMV, HV, T. gondii and B. ceti were negative in both animals, with the only exception of a positive result for morbilliviral genome in the lung tissue from the second dolphin.

The extracted organic material (EOM%) and the levels of OC pollutants (HCB, DDTs, PCBs) found in tested tissues of the two individuals, expressed in ng/g dry weight (d.w.), are reported in Table 1. The highest levels of OC contaminants were detected in the blubber, followed by the liver and the skeletal muscle from both dolphins. Total PCBs were the most representative contaminants in every tissue tested, followed by total DDTs and HCB.

**Table 1:** Extracted organic material (EOM %) and organochlorine contaminants (HCB, DDT e PCB) in sampled tissues from both cases, expressed in ng/g dry weight (d.w).

	**Case 1**			**Case 2**		
**Tissue**	**Fat**	**Liver**	**Muscle**	**Fat**	**Liver**	**Muscle**
MOE%	83.00	6.40	7.20	68.40	6.80	2.50
HCB	374.11	11.55	4.57	529.69	145.42	5.71
op'DDE	2340.98	85.40	68.22	1450.43	378.78	16.07
pp'DDE	118022.03	4100.94	3488.71	66382.60	17019.69	631.22
op'DDD	1855.52	76.07	68.43	1885.16	491.24	22.05
pp'DDD	5038.76	101.08	111.37	3856.72	864.42	26.56
op'DDT	1367.25	37.93	32.48	663.25	116.35	N.R.
pp'DDT	3598.91	139.91	118.70	2218.85	547.29	18.34
DDT tot ps	132223.46	4541.32	3887.91	76457.00	19417.77	714.24
95	1459.30	47.74	46.21	1667.39	386.36	18.03
101	1892.06	74.03	60.86	2360.58	614.11	24.33
99	122.39	6.01	4.95	302.82	78.73	N.R.
151	2746.77	127.55	100.17	1676.30	475.87	20.53
144+135	1232.08	84.29	63.58	1567.75	427.95	17.96
149+118	5550.21	280.81	201.20	8737.28	2584.80	109.70
146	6244.91	309.20	238.65	4395.71	1314.41	50.86
153	31999.23	1726.79	1303.29	18816.73	6221.82	244.70
141	1773.63	103.92	73.95	1142.57	389.35	19.27
138	18010.76	896.48	723.01	10848.01	3378.78	146.43
178	3002.38	190.01	136.13	1581.54	557.97	22.81
187	13294.91	869.00	611.41	6552.05	2346.33	105.31
183	3581.43	238.67	172.27	1998.67	744.05	32.09
128	1304.81	55.12	51.34	1175.78	318.15	12.45
174	3628.64	251.65	178.68	2556.83	854.07	35.41
177	3290.21	193.04	145.90	1748.49	536.93	25.78
156+171+202	1315.97	69.99	98.92	1606.48	518.43	22.48
172	1083.24	68.96	49.88	883.13	291.49	12.06
180	18103.99	1154.93	874.03	9373.23	3432.07	147.10
199	105.79	6.58	7.60	N.R.	23.67	N.R.
170	10902.57	595.51	485.23	6079.63	1866.82	95.44
196	2943.43	211.76	155.31	1712.31	622.90	25.75
201	2185.93	154.41	115.72	1256.74	455.29	20.94
195	962.53	54.52	45.72	557.84	181.61	35.41
194	1110.95	79.84	63.17	842.22	321.43	17.52
206	168.48	18.65	10.32	N.R.	78.01	13.17
PCB tot ps	138016.60	7869.46	6017.50	89440.10	29021.39	1275.55

These findings are consistent with those reported in tissue samples from striped dolphins stranded in the same areas of the Western Mediterranean basin, which usually have PCBs as major contaminants (17). In both cases herein examined, the levels of DDTs and PCBs were similar in the blubber, with high levels of these OC compounds being also found in hepatic and skeletal muscle tissues. Organochlorine pollutants are known to exert prominent immunotoxic effects and reproduction function impairment, as these lipophylic contaminants are powerful endocrine disrupting chemicals (18, 19); the tissue levels determined in case 1 were higher than those considered to be “homeostatic” for the Mediterranean Sea (16). Indeed, a CAN value higher than 0.47 denotes a compromised toxicological condition (16) and, thereafter, the CAN value of 0.974 found in dolphin 1 strongly supports an immunosuppression condition in this animal; conversely, animal 2, exhibiting a CAN value of 0.267, could not be classified as potentially affected by a toxicological hazard. With regards to the role of morbilliviral infection as a causative and/or contributing factor to both lesions development and the stranding and death of the second dolphin, the detection of morbilliviral genome in the lung parenchyma could argue in favor of a “non-active/productive infection.” Morbilliviruses are known to be highly lymphotropic and strongly immunosuppressive pathogens across a number of susceptible terrestrial and aquatic mammal species, and a synergistic pathogenic interplay has been repeatedly suggested between aquatic mammal morbilliviruses and immunotoxic environmental pollutants like PCBs. However, caution is advised in the interpretation of the aforementioned results.

The Authors report herein the first identification and description of CePV infection in striped dolphins found stranded along the Italian coastline. This is supported by clear-cut morpho-pathological and ultrastructural evidences of CePV involvement in the etiology of the skin lesions observed in the two herein investigated striped dolphins, with this information being ultimately corroborated by the genetic characterization of the causative poxviral agent. This appears to be of relevance, among others, also in relation to the paucity of pathological descriptions of CePV infection among Mediterranean cetaceans. The concurrent detection of high tissue levels of OC compounds supports the putatively occurring synergistic interplay between CePV, and several immunotoxic environmental pollutants (5, 7). However, this potentially “dangerous relationship” as well as the putative role of other stressors, including viral and non-viral pathogens, warrant further investigation.

## Author contributions

CC and GF participated in the experimental procedures and data analysis, and contributed equally to manuscript writing. GC, AC, and ER participated in performing data analysis and manuscript writing for the sequence comparison and phylogenetic analysis. LM, MS, FG, and FS gave contributions in the acquisition of necropsy, microbiological, and toxicological data for the work. GD and GT supervised the different phases and manuscript writing. All authors reviewed and agreed on the current version of the manuscript.

### Conflict of interest statement

The authors declare that the research was conducted in the absence of any commercial or financial relationships that could be construed as a potential conflict of interest.

## Supplementary material

Figure S1 Numerous round, up to 25 μm, eosinophilic glassy structures (intracytoplasmic inclusion bodies) surrounded by a clear halo (Guarnieri bodies). Scale bar = 100 μm.

Figure S2 Electron micrograph of skin lesion sample showing negatively stained brick viral particle of ≈150–310 nm, consistent with Cetacean poxvirus. Scale bar = 100 nm.
