# Supplementary figures and images for: Cetacean Poxvirus in Two Striped Dolphins (Stenella coeruleoalba) Stranded on the Tyrrhenian Coast of Italy: Histopathological, Ultrastructural, Biomolecular, and Ecotoxicological Findings
Source: Front Vet Sci. 2018 Sep 11;5:219. doi: 10.3389/fvets.2018.00219 (PMC6141780; doi:10.3389/fvets.2018.00219)

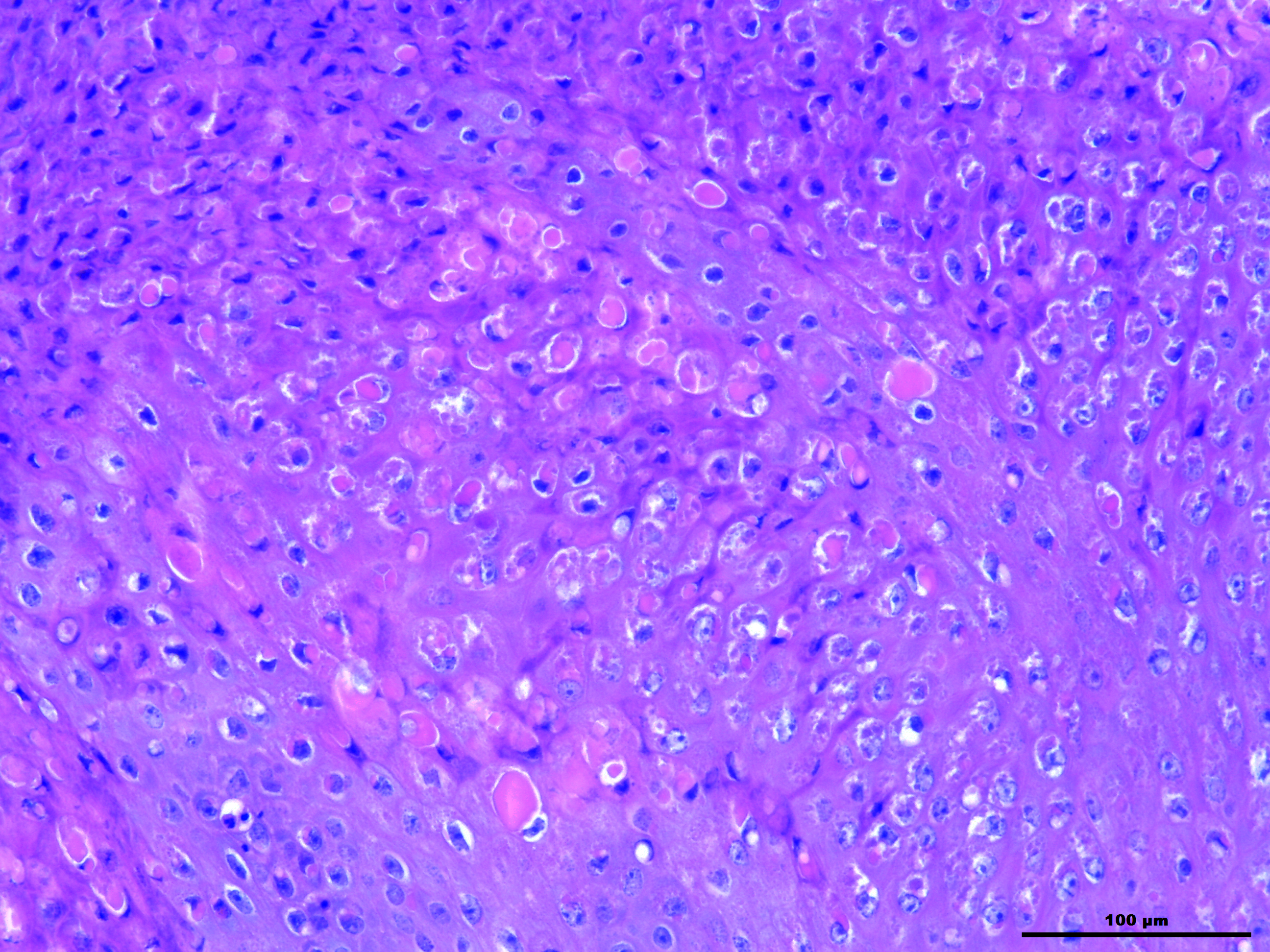

Supplement: Figure S1 — Numerous round, up to 25 μm, eosinophilic glassy structures (intracytoplasmic inclusion bodies) surrounded by a clear halo (Guarnieri bodies). Scale bar = 100 μm. [file Image_1.TIF]

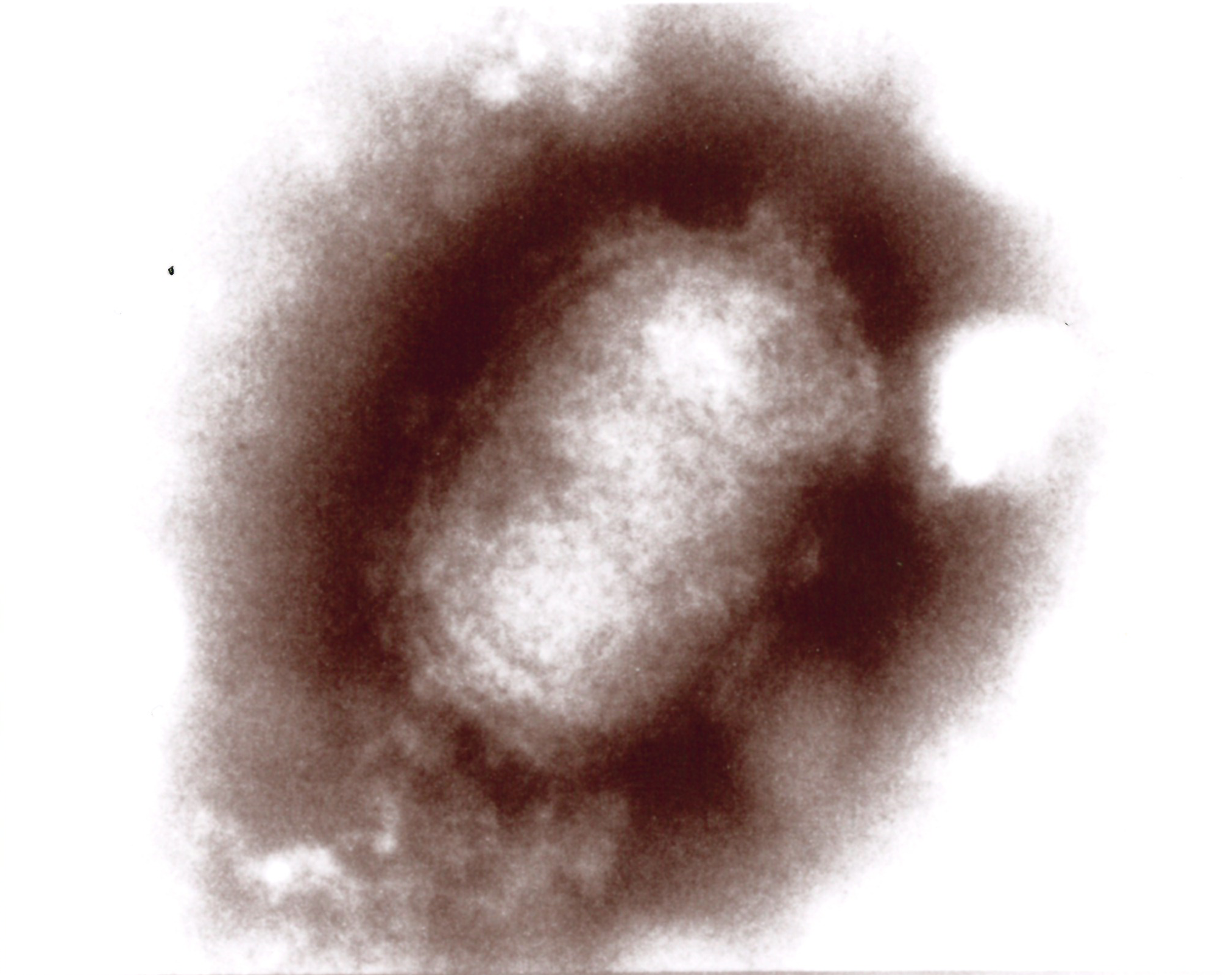

Supplement: Figure S2 — Electron micrograph of skin lesion sample showing negatively stained brick viral particle of ≈150–310 nm, consistent with Cetacean poxvirus. Scale bar = 100 nm. [file Image_2.TIF]
